# Genome-wide functional analysis using the barcode sequence alignment and statistical analysis (Barcas) tool

**DOI:** 10.1186/s12859-016-1326-9

**Published:** 2016-12-23

**Authors:** Jihyeob Mun, Dong-Uk Kim, Kwang-Lae Hoe, Seon-Young Kim

**Affiliations:** 1 Korea Research Institute of Bioscience and Biotechnology (KRIBB), Personalized Genomic Medicine Research Center, Daejeon, Republic of Korea; 2 Department of Functional Genomics, University of Science and Technology, Daejeon, Republic of Korea; 3 Korea Research Institute of Bioscience and Biotechnology (KRIBB), Aging Research Center, Daejeon, Republic of Korea; 4 Graduate School of New Drug Discovery and Development, Chungnam National University, Yusong-gu, Daejeon, South Korea

**Keywords:** Barcode sequencing, Trie data structure based imperfect matching algorithm, Pooled library screen analysis, shRNA, sgRNA, Barcoded yeast deletion strains

## Abstract

**Background:**

Pooled library screen analysis using shRNAs or CRISPR-Cas9 hold great promise to genome-wide functional studies. While pooled library screens are effective tools, erroneous barcodes can potentially be generated during the production of many barcodes. However, no current tools can distinguish erroneous barcodes from PCR or sequencing errors in a data preprocessing step.

**Results:**

We developed the Barcas program, a specialized program for the mapping and analysis of multiplexed barcode sequencing (barcode-seq) data. For fast and efficient mapping, Barcas uses a trie data structure based imperfect matching algorithm which generates precise mapping results containing mismatches, shifts, insertions and deletions (indel) in a flexible manner. Barcas provides three functions for quality control (QC) of a barcode library and distinguishes erroneous barcodes from PCR or sequencing errors. It also provides useful functions for data analysis and visualization.

**Conclusions:**

Barcas is an all-in-one package providing useful functions including mapping, data QC, library QC, statistical analysis and visualization in genome-wide pooled screens.

**Electronic supplementary material:**

The online version of this article (doi:10.1186/s12859-016-1326-9) contains supplementary material, which is available to authorized users.

## Background

Barcode-seq is a next-generation sequencing (NGS) technique that reads genome-integrated artificial sequences called barcodes that specifically mark biological materials, such as cells or genes, with unique sequences [1]. Having a unique barcode facilitates tracking materials of interest in genome-wide functional screens as well as the identification of drug targets or disease-associated genes [2]. Currently, barcode-seq is used in several genome-wide screening tools, including shRNAs for gene knock-down [3], sgRNAs for genome editing [4] and barcoded yeast deletion strains in *Saccharomyces cerevisiae* and *Schizosaccharomyces pombe* for gene knock-down or knock-out [1, 5, 6]. The use of these tools for loss-of-function studies allows the discovery of relationships between genes and a specific environment without prior knowledge. Thus, barcode-seq is an effective system for genome-wide screening studies to comprehensively understand biological systems.

Previously, barcoded screening was mostly performed using microarrays. However, barcode-seq has recently become more popular than microarrays due to its sensitivity, wider dynamic range and limits of detection [1]. Moreover, by multiplexing, barcode-seq can now be run more cheaply than a barcode microarray [7]. However, there are many steps during barcode-seq that can generate errors: chemical synthesis of oligonucleotides, PCR amplification and NGS to name a few [1]. Thus, it is necessary to consider all kinds of possible errors when performing barcode-seq experiments.

With more researchers using barcode-seq for genome-wide screens, needs for tools for data preprocessing and statistical analysis are increasing. For data preprocessing, a few tools exist including BiNGS!LS-seq [8] based on bowtie [9], shALIGN [3] and edgeR package [10, 11] of Bioconductor [12]. (Although edgeR is commonly famous for differential expression analysis, it also supports a function of barcode read alignment through ‘processAmplicons’ method allowing mismatches and small shifts for start positions of barcodes. Therefore, edgeR is being used as barcode-seq alignment in shRNA data analysis [13].) For the analysis of shRNAs or sgRNAs, the existing tools allow mismatches and small shifts to include sequencing or PCR errors [14, 15]. Although accommodating sequencing errors increases mapped read counts, it is also possible to gain read counts of erroneous barcodes, which may work for off-targets [16–18]. Therefore, when mapping with imperfect matching, filtering erroneous barcode counts should be accompanied, but no tool currently provides a function to filter out erroneous reads.

Here, we present Barcas software as a comprehensive tool for the analysis of multiplexed barcode-seq data. We employ the trie data structure for sequence mapping to efficiently survey diverse situations including mismatches, indels and barcode frame-shifts. We also developed a novel process of identifying and filtering out erroneous barcodes that are different from the originally designed sequences. Another novel function of Barcas is the barcode library QC function to identify a group of barcodes with high sequence similarity as a potential source of errors in mapping. Moreover, Barcas performs all the steps of barcode-seq data analysis in a streamlined manner and also provides diverse graphs and tables that allow for an intuitive understanding of the results and compatibility with other tools.

## Methods

### Implementation

Each shRNA, sgRNA or yeast deletion strain is designed to knock-down or knock-out of a gene. To find target genes for a specific phenotype or validate target genes, shRNAs, sgRNAs or yeast deletion strains are used in pooled library screen analysis (Additional file 1: Figure S1).

### Barcode-seq data

**Fig. 1:**
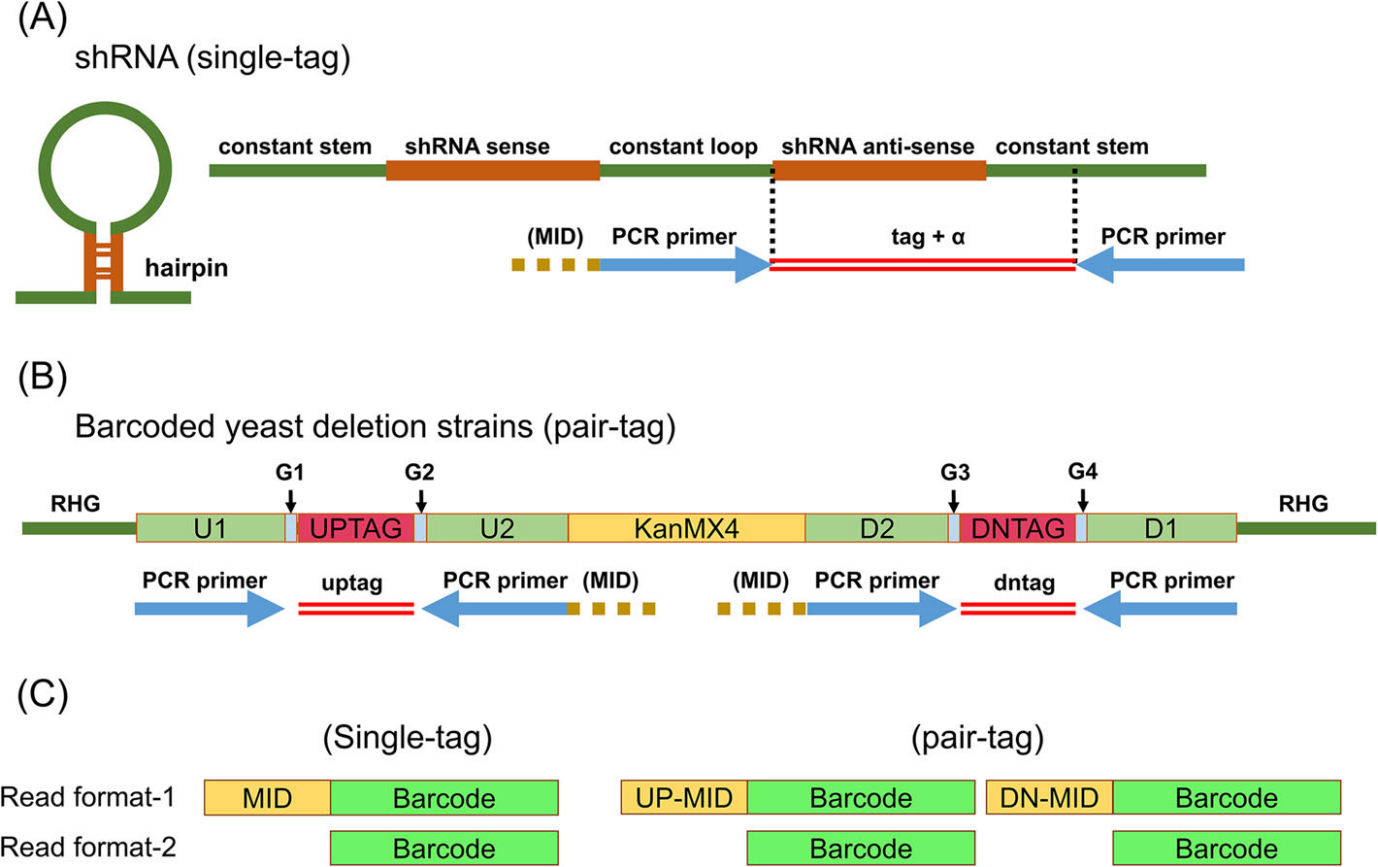
Barcode-seq data. **a** Barcode DNAs of shRNAs. Sense or anti-sense strands of shRNAs are generally used for barcodes (barcode sequences and shRNA sequences are the same.). However, sometimes, barcodes are separately inserted to identify shRNAs (barcode sequences and shRNA sequences are different.), such as the Cellecta library; **b** Barcode DNAs of yeast deletion strains. For a gene, there are two barcodes (‘UP’ and ‘DN’) in yeast deletion strains; **c** Two read formats for single-tag and pair-tag in barcode-seq data. In a barcode sequence region, it also contains a universal primer sequence

A barcode DNA is generally composed of a universal primer sequence and a unique barcode sequence. Barcode-seq data are a collection of sequences of barcode regions that are sequenced by NGS after amplification by PCR using the universal primers (Fig. 1). As an example, a shRNA anti-sense sequence is a barcode sequence in shRNAs [3]. Normally, there is one barcode for each shRNA or sgRNA, while there are two barcodes (called ‘UP’ and ‘DN’) for each barcoded yeast deletion strain (Fig. 1b). We use the term ‘single-tag’ for libraries having one barcode for a target region and ‘pair-tag’ for libraries having two barcodes for a target region. For example, shRNAs and sgRNAs are ‘single-tag’ while yeast deletion strains are ‘pair-tag’. Although shRNAs and sgRNAs have several barcodes in a gene, each barcode is designed to target different sequences in a gene. On the contrary, each yeast deletion strain has its gene sequence replaced by an artificial sequence containing two barcodes on both sides. Therefore, mapped read counts between two barcodes are expected to be similar. For multiplexed sequencing in a single lane, a Multiplexed ID (MID) can be added to the 5′end of a primer. In general, FASTQ files are generated after sequencing (Fig. 1c).

### Mapping algorithm

Barcas maps sequenced reads based on the trie data structure for fast and efficient imperfect matching. The trie data structure is composed of a root, internal nodes and leaves (end nodes). Starting from a root, a node is connected with its high level node and several low level nodes or leaves. Here, Barcas modified trie data structure to accept imperfect matching for barcode-seq data (Additional file 1: Table S1). A node or leaf corresponds to a base of a barcode sequence. Barcas thus constructs a trie data structure from the barcode library sequences and maps input reads to sequences in the trie. The trie data structure based imperfect matching algorithm facilitates the comparison of sequences of different lengths quickly and compares input reads to all the sequences of the reference library considering mismatches and indels. From the root node, Barcas compares bases between library sequence and read sequence considering all cases, match, mismatch, insertion and deletion (every node within accepted error count). Barcas also uses primer sequences to fix shifts of a barcode. If there are any indels while mapping primer sequences, Barcas considers the indels as a shift for a barcode sequence. And, the program adjusts a start position of a barcode sequence every read. Thus, by employing the trie data structure based imperfect matching algorithm, it generates more accurate mapping results in comparison to currently available tools and does so with less processing time. The gain in mapping accuracy and speed of the trie structure requires extra memory, as the trie structure consumes more memory as a sequence gets longer due to recursive function. In the Java language, a node corresponds to an object and the maximum size of a tree data structure is ∑_0_^*n*^ *b* ^*n*^ × s bytes (b: the number of bases such as A, G, C, and T; n: sequence length; s: object size). For example, Barcas needs about 350 MB memories for uploading Yusa library which barcode sequence is 19-bp and the number of sequences is 87,437 [19]. Thus, while the trie data structure is impractical for references of long sequences, it is applicable to mapping of barcode sequences which are normally shorter than 50 bps. With the trie data structure, Barcas maps a read to a MID sequence (optional), primer sequence (optional) and a barcode sequence linearly or from a user defined start position. It checks every base of a read from 5′ to 3′ serially, and decides whether the read is mapped or not and how many bases are mapped in a sequence considering mismatches and indels. Lastly, the program selects uniquely mapped reads, but counts of ambiguous reads are also saved to measure data quality.

### Analysis

Barcas was developed to find over-represented or depleted target barcodes through comparing intensity between case samples and control samples. And, a barcode is commonly meant as a gene. With case and control samples, each mapped barcode count is first summed of 1 to make more than zero and normalized as ratio by the total count in a sample and calculated to a mean for replicates. After that, the program filters barcodes in which both case and control samples have lower mapped counts than a user defined barcode count (the default filtering count is 100). Next, fold-change score and Z score are calculated for each barcode based on the growth inhibition score (GI) [20].

$$ z = \kern0.5em \left(x- mean\right)/\left( standard\  deviation\right) $$

For shRNA and sgRNA data which barcodes belong to a specific gene, barcodes are clustered using fold-change (fc) score and Z score in a group. And, clustered sets are filtered to remove off-target when the number of members in a clustered set is less than quarter of total in a group. After that, clustered-level fold-change score and Z score are also calculated.

Using Z scores, Barcas ranks barcodes and clustered sets and provides a scatterplot graph to help users to identify significantly depleted or enriched target genes. In the case of time-series data, Barcas also provides heat-maps of time-dependent barcode intensity variation using fc-scores by times.

### Workflow for analyzing barcode-seq data

**Fig. 2:**
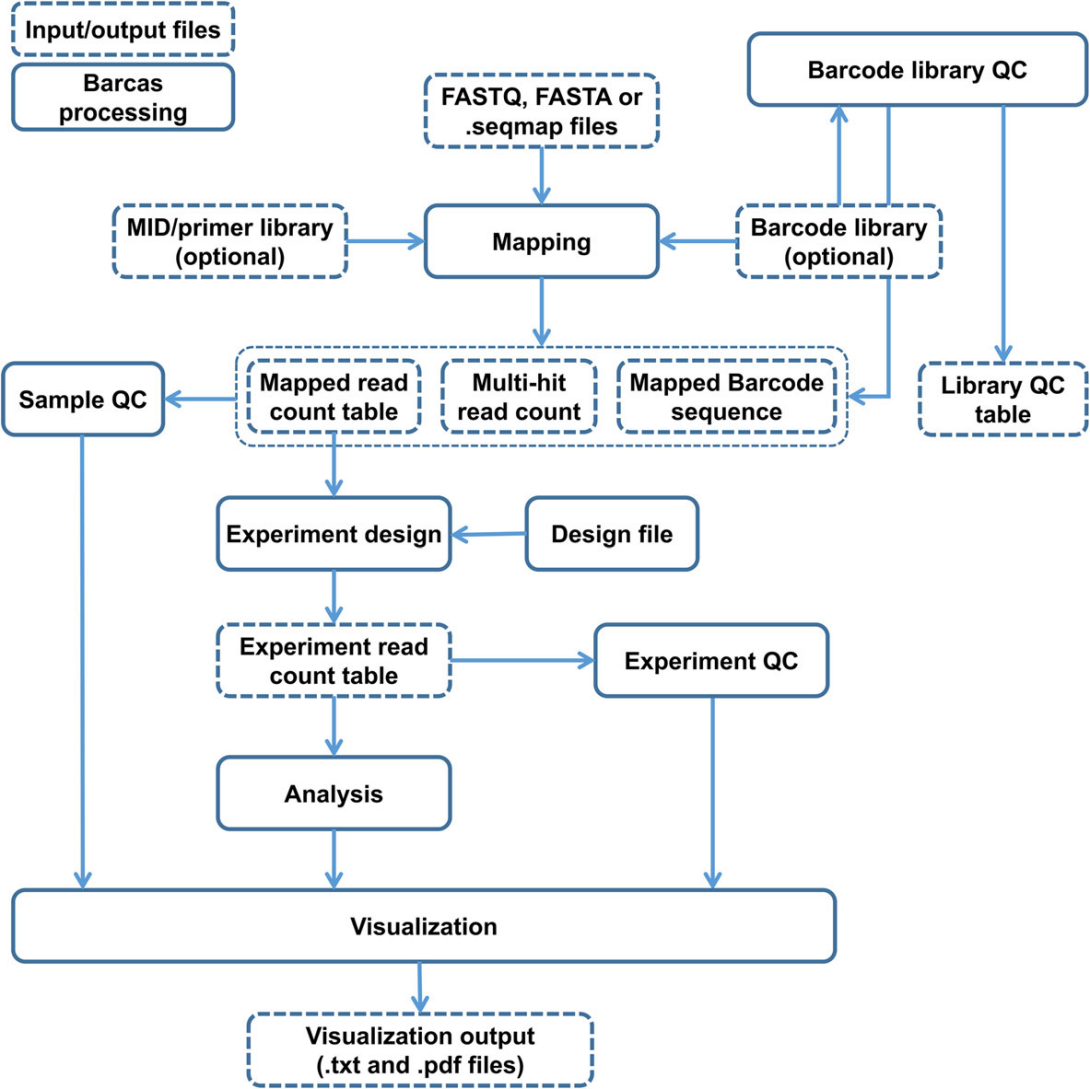
Barcas workflow. Barcas consists of five modules, Mapping, library QC, data QC, statistical analysis and visualization. For compatibility with other tools, the program generates text-based output files in each step

The analysis workflow of Barcas includes mapping, library QC, data QC, statistical analysis and visualization processes (Fig. 2). In each step, the program exports simple text files as outputs for use in other tools. Thus, users can take advantage of any of Barcas modules for their convenience.

#### Input

Barcas accepts FASTQ, FASTA or seqmap files as an input. A seqmap file is a plain text file that consists of unique sequences and their read counts and was designed to reduce mapping time in Barcas because many identical short reads exist in barcode-seq data. FASTQ or FASTA files are first converted into seqmap format for further processing.

#### Mapping

While most barcode sequencing data are basically composed of a MID and a barcode sequence with a primer sequence (Fig. 1c), the specific composition and sequence length of each library differs greatly. For example, lengths of MID sequence in the Cellecta library vary from 9-bp to 17-bp. In the TRC shRNA library, while most barcodes are 21-bp long, several barcodes that are 20-bp long are also included. In another case, while barcodes for shRNAs and sgRNAs are designed as single-tag, barcoded yeast deletion strains are designed as pair-tag. Additionally, erroneous barcode sequences may be generated at any stage of experiments including barcode synthesis, library construction, library strain maintenance, PCR and sequencing steps.

To support mapping for all types of barcode-seq data, many factors should be considered: the existence of MIDs, dynamic sequence lengths, dynamic start position of a barcode, mismatches, position shifts of barcodes, indels and elimination of erroneous barcode counts. We designed Barcas to handle all of the above mentioned conditions flexibly. Additionally, Barcas supports both perfect and imperfect matching during mapping and barcode library QC steps. No other previously developed programs support the above mentioned diverse situations. For example, while the edgeR package allows position shifting for barcode mapping, it uses a unified start and end position regardless of dynamic sequence lengths and does not support indel mapping.

#### Barcode library QC

A barcode library is a collection of tens to hundreds of thousands of short sequences (Additional file 1: Table S1, information of public barcode libraries) which are subject to errors such as mutations during barcode synthesis or library strain maintenance and insertion into wrong genomic regions in the case of yeast barcode libraries, etc. As barcode sequences are short, many barcode sequences in the library have very similar sequences (less than two base pair difference). In this case, random mutations during barcode ﻿maintenance﻿,﻿ barcode insertion into a cell, ﻿PCR or sequencing by NGS can generate mutated barcodes that can be mapped to wrong (but similar) target genes (off-target effects). Barcas provides three functions for library QC to fix the above mentioned errors. Firstly, the program checks whether a barcode is an erroneous one or not, and if erroneous, removes it from mapped reads optionally. Second, the program identifies erroneous barcodes and possibly fixes them to make an error-free library. Third, the program computes all pairwise similarities among sequences in a library, and reports a list of barcodes that should be analyzed carefully due to high sequence similarity. By providing these three functions, the program helps users to choose an appropriate library and barcodes for a study and increases the mapping ratio without erroneous barcodes.

#### Data QC

Barcas shows counts and the percentage of mapped reads, counts of filtered reads by multiple mapping, correlations between up and down tags for pair-tag experiments, and the read count ratio of barcodes in a sample.

#### Analysis

Barcas analyzes data based on comparison between two-conditions (possibly) with multiple time points. To discover significant target genes, it generates z-scores, fc-scores and ranks of barcodes and genes between controls and cases and fc-scores between different time points.

#### Visualization

Barcas shows analysis results, the data QC and the barcode library QC as tables or graphs of several types. Search for a specific barcode is supported in both tables and graphs to help users identify barcodes of interest. Users can export tables as a tab-delimited text files and graphs as a PDF (.pdf) files.

## Results and Discussion

### Sequencing or PCR errors vs. erroneous barcodes

While barcode-seq data should ideally be mapped by perfect matching, systematic errors, such as errors that occur during PCR amplification or random mutations during strain maintenance, can lead to erroneous (or mutated) barcode sequences that are not mapped by perfect matching. To overcome such systematic errors, many existing tools allow mismatches or shifts during mapping.

**Fig. 3:**
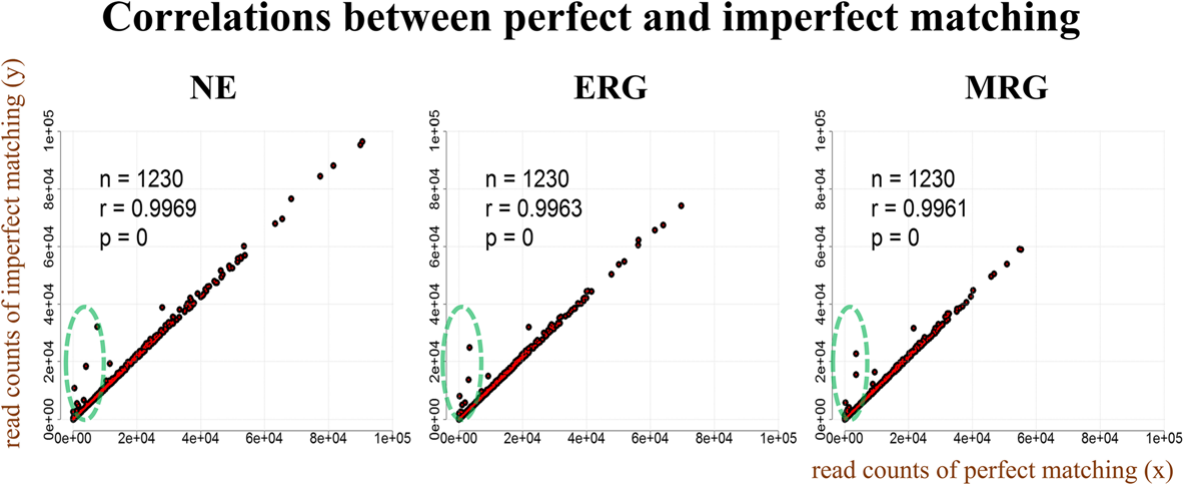
Comparison between perfect and imperfect matching. With only forward barcode sequences, reads were mapped to 1,230 shRNA sequences from the TRC library. Total read counts are 22,497,317 (NE), 17,003,721 (ERG) and 22,573,358 (MRG). In the imperfect matching, at most, two base pair errors, mismatches and indels, were allowed. Compared to perfect matching, imperfect matching mapped an additional 3.64%, 3.55% and 2.59% reads in NE, ERG and MRG samples. While most barcodes showed similar mapped counts between perfect matching and imperfect matching, several barcodes were mapped much better in imperfect matching

Here, we distinguish two types of errors for barcode sequences: errors during sequencing with the originally correct barcode, and errors in the barcode itself (possibly by erroneous barcode synthesis, mutations during library maintenance, and erroneous incorporation of the barcode into the genome in the case of yeast strains, etc.). While sequencing errors generally have negligible effects on the increases in the mapped counts by imperfect mapping, erroneous barcodes have huge effects on the resulting mapped counts by imperfect mapping (Fig. 3).

As examples, we show the results of the analyses of two public data sets. The first data set consists of human shRNA-seq samples in neuroepithelial (NE), early radial glial (ERG) and mid radial glial (MRG) cells [13]. Each sample was obtained from a sub-pool of the human 45K shRNA pool containing 1,230 shRNAs from the RNAi Consortium (TRC). Only forward sequences of control samples (24-h) were used. When we compared mapped barcode counts between perfect and imperfect matching, we identified 25 erroneous barcodes (2.03%) of which mapping counts increased hugely compared to their original barcode sequences (Fig. 3; Additional file 1: Figure S2 and Table S2, standard for determining erroneous barcodes).

Overall, the additional read counts were 56,015 (0.25%), 45,321 (0.27%) and 40,450 (0.18%) from 21, 17 and 13 erroneous barcodes, and 762,929 (3.39%), 558,448 (3.28%) and 543,790 (2.41%) from sequencing or PCR errors in NE, ERG and MRG, respectively.

**Fig. 4:**
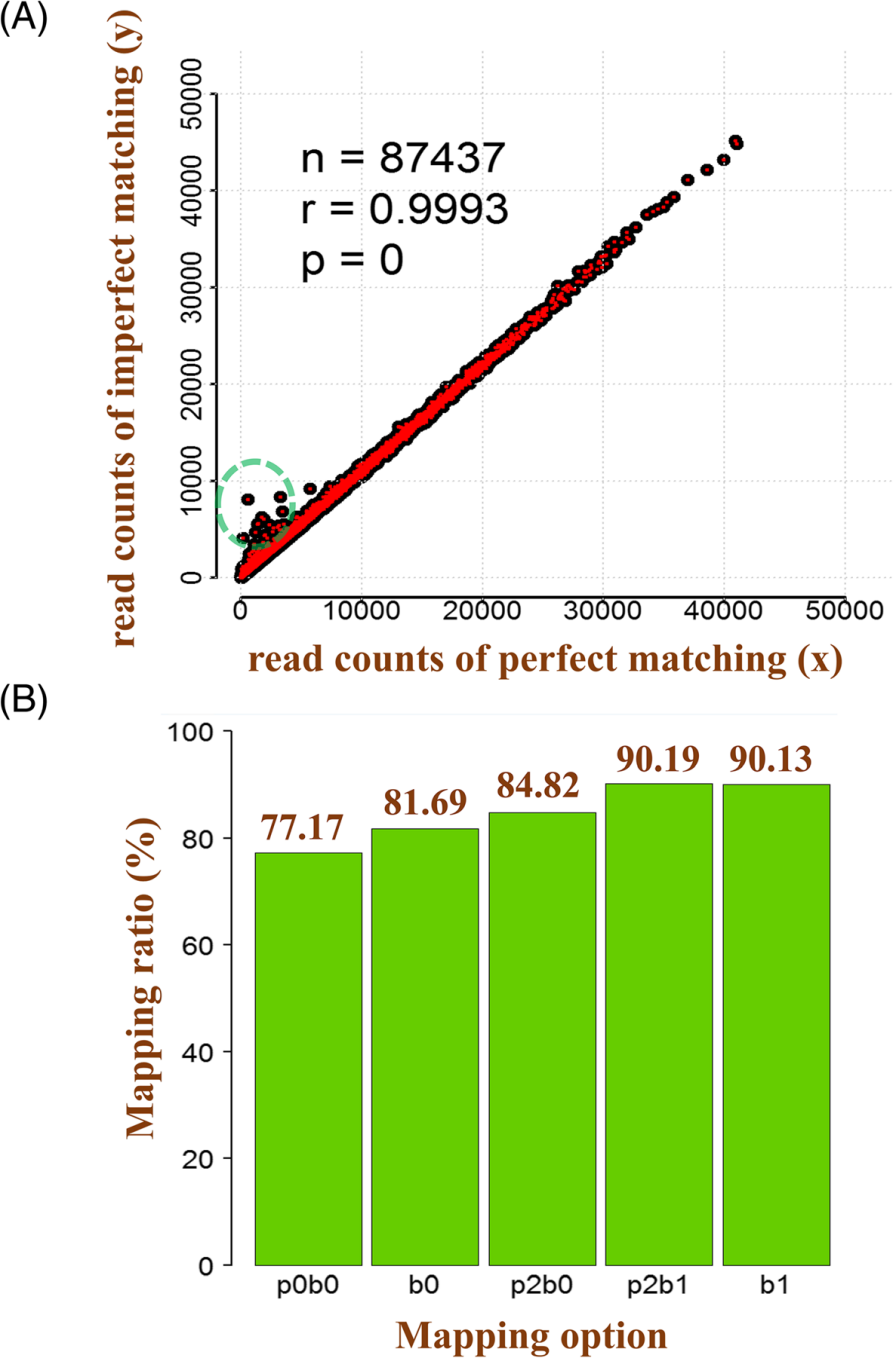
Mapping results from the yusa library. **a** Comparison between perfect and imperfect matching. The yusa library had 72 erroneous barcodes with one base pair error; **b** Comparison of mapping between with and without primer sequences. Comparison between p0b0 and p2b0 shows that there were erroneous sequences in primer sequences for various reasons. Although Barcas supports shifts through primer sequence mapping, when there are small indels in primer sequences, it showed similar results between p2b1 and b1. (‘p’ is a primer; ‘b’ is a barcode; The number is allowed error base counts)

The second data set was sgRNA-seq data from mouse embryonic stem cells [19]. When we mapped a total of 288,215,818 reads to 87,437 sgRNAs from the yusa library, we identified 72 erroneous barcodes (0.08%) with one mismatch or indel. Here, the additional read counts were 81,355 (0.03%) from 72 erroneous reads and 15,406,665 (5.34%) from sequencing or PCR errors (Fig. 4).

Erroneous barcodes identified from the above two data sets showed mostly mismatched as opposed to deletions and a few insertions. In barcoded deletion strains, erroneous barcodes would have negligible effects on the deletion of the corresponding genes. However, for shRNA interference experiments in which perfect sequence matching is necessary for gene knock-down, erroneous barcodes are better to be filtered out as they are unlikely to knock-down their target genes. Barcas provides an option to filter out erroneous barcodes from subsequent analysis.

### Barcode library QC

Although Barcas supports filtering erroneous barcode counts, it can’t be distinguished when there are barcodes similar each other in a pool and someone is changed to another sequence by mutation or sequencing errors. Barcodes in a library should have distinct sequences to one another at least more than two bases. However, due to the large size of a barcode library (Additional file 1: Table S1) and restrictions in designing a specific target region for a gene, designing a library of barcodes with maximally dissimilar sequences may be difficult. Barcas provides a function to calculate the pairwise similarities between barcodes in a library to estimate the potential risks of mutation, sequencing or PCR errors. When we surveyed the pairwise similarities among barcodes in TRC, Cellecta, yusa, GeCKOv2 and barcoded deletion mutant strains (Tables 1 and 2; Additional file 1: Table S1), we found that many libraries have a significant proportion of barcode sequences that differ by only one base. To avoid off-target problems by similar barcode sequences, it is better not put similar barcodes in a pool in experimental design step.

**Table 1:** Barcode count having similar pairs within one bas

Library	Static sequence length comparison	Dynamic sequence length comparison
GeCKOv2.Human.A	517 (0.81%)	538 (0.84%)
GeCKOv2.Human.B	437 (0.77%)	441 (0.78%)
GeCKOv2.Mouse.A	736 (1.12%)	755 (1.14%)
GeCKOv2.Mouse.B	850 (1.39%)	860 (1.41%)
.yusa	517 (0.59%)	3,944 (4.51%)
Cellecta.Human.M1	0 (0 %)	412 (1.5%)
Cellecta.Human.M2	0 (0 %)	398 (1.45%)
Cellecta.Human.M3	0 (0 %)	410 (1.49%)
TRC	790 (1.28%)	1,909 (3.10%)
*Cerevisiae*	0 (0 %)	0 (0 %)
*Pombe*	0 (0 %)	0 (0 %)

Dynamic sequence length comparison and static sequence length comparison: if comparing sequences between ‘AGCT’ and ‘ACTA’, then the mapped sequence patterns are ‘AGCT-’ and ‘A-CTA’. Based on ‘AGCT’, the sequence has similar pairs within two bases in static sequence length comparison and one base in dynamic sequence length comparison. Dynamic sequence length comparison method doesn’t consider backward hyphen because the sequence already finished. Barcas uses dynamic sequence length comparison while mapping reads to library sequences

**Table 2:** Designed barcode patterns of libraries

Library	Static sequence length comparison				Dynamic sequence length comparison			
	Count	intra	Intra & inter	inter	Count	intra	Intra & inter	inter
GeCKOv2.Human.A	517	27 (5.22%)	3 (0.58%)	487 (94.20%)	538	30 (5.58%)	3 (0.56%)	505 (93.87%)
GeCKOv2.Human.B	437	0 (0.0%)	0 (0.0%)	437 (100.0%)	441	0 (0.0%)	0 (0.0%)	441 (100.0%)
GeCKOv2.Mouse.A	736	8 (1.09%)	0 (0.0%)	728 (98.91%)	755	10 (1.32%)	0 (0.0%)	745 (98.68%)
GeCKOv2.Mouse.B	850	6 (0.71%)	0 (0.0%)	844 (99.29%)	860	6 (0.7%)	0 (0.0%)	854 (99.3%)
.yusa	517	2 (0.39%)	0 (0.0%)	515 (99.61%)	3,944	3,415 (86.59%)	48 (1.22%)	481 (12.20%)
Cellecta.Human.M1	0				412	0 (0.0%)	0 (0.0%)	412 (100.0%)
Cellecta.Human.M2	0				398	0 (0.0%)	0 (0.0%)	398 (100.0%)
Cellecta.Human.M3	0				410	0 (0.0%)	0 (0.0%)	410 (100.0%)
TRC	790	18 (2.28%)	0 (0.0%)	772 (97.72%)	1,909	1,045 (54.74%)	12 (0.63%)	852 (44.63%)

(Intra: similar barcodes are in the same gene. Inter: similar barcodes have different gene. Most libraries have more inter barcodes than intra barcodes within one base distinction

### Comparison of performance

We compared the mapping speed and accuracy of Barcas with two other tools, bowtie v.1.1.2 and edgeR package (R version 3.2.5 and limma version 3.26.9) (Additional file 1: Table S3, comparison environment, used options for three programs and mapping result). We used the barcode-seq data of 215 million reads from barcoded deletion strains of *Schizosaccharomyces pombe* [5]. We used 45-bp seuences, primer sequence and barcode sequence (20-bp), as a barcode library. As a result, Barcas generated 8% more reads than the edgeR package and bowtie. For mapping speed, Barcas was about 1.7 times faster than bowtie and 13 times faster than edgeR (Additional file 1: Table S3).

### Visualization

**Fig. 5:**
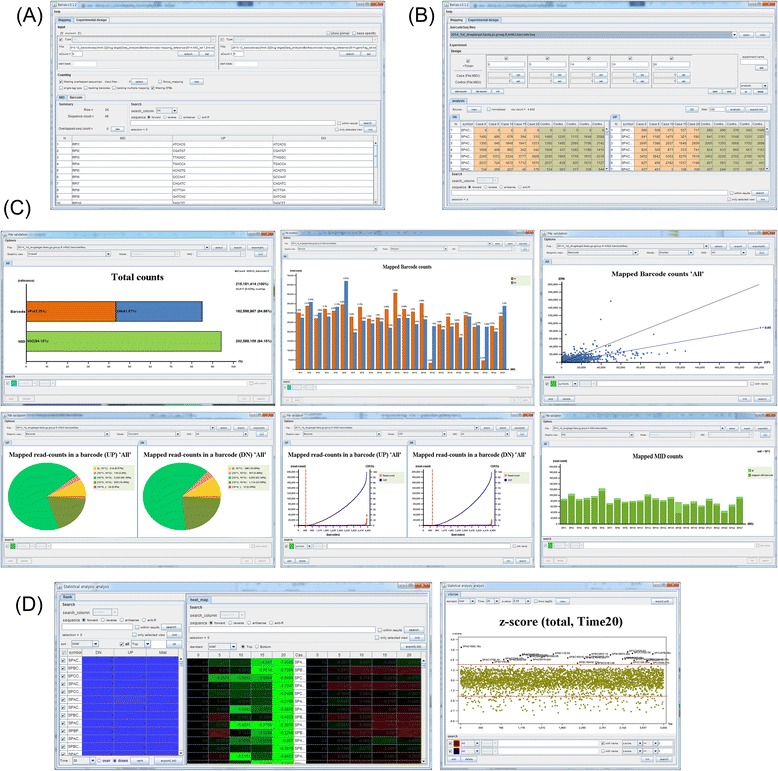
Visualization of Barcas. **a** Mapping; **b** Designing experiments; **c** Data QC; **d** Statistical analysis. In Barcas, whole steps of analyzing barcode-seq data are progressed by graphic interface

Barcas is an all-in-one program written in Java. All steps are executed in the graphical user interface (Fig. 5). For reporting the mapping results, Barcas generates a table of mapped read counts with multiple hits and mapped read sequences of barcodes including erroneous barcode information (Fig. 5a). With mapped read counts, Barcas runs statistical analysis and generates tables of ranks of each barcode, graphs of enrichment scores and a time-dependent heat-map of enrichment scores (Fig. 5d). With multiple searching systems, genes of interest can be easily identified in the tables and graphs.

## Conclusions

For pooled library screen analysis, it is important issue to filter off-targets. Currently almost tools support a function to filter off-targets in a statistical analysis step only. Otherwise, Barcas supports a function of filtering off-targets in a data preprocessing also. Barcas has many useful features for preprocessing of barcode-seq data. It uses the trie data structure based imperfect matching algorithm which gives more correct results in barcode mapping and efficient library QC with a small cost of more memory usage. For mapping, any type of barcode-seq data can be used in the program as it allows for multiplexing, dynamic sequence lengths and imperfect matching. Through library QC, users can distinguish erroneous barcodes from random sequencing errors, and check for the similarities of barcodes in the library. These functions could help to filter out off-targets while increasing the mapping rate. Barcas supports basic statistical analysis of barcode-seq data and gives similar result with existing tools, such as RIGER [21] and Chris Vakoc [22]. However, Barcas currently lacks several methods and tools, such as multiple-condition comparisons [23] and utilization of metadata [24]. For compatibility with other tools, Barcas is modularized and exports text-based results in any of the analysis steps. Barcas is a graphical user interface based all-in-one package for analyzing barcode-seq data. We hope it will be useful for researchers conducting genome-wide barcode-seq experiments for various purposes.

## Additional file

Additional file 1: Figure S1. Pipelines of pooled library screen analysis. **Table S1.** Public library sets of shRNA, sgRNA and deletion mutant strains. **Figure S2.** A list of wrong barcodes from 1,230 shRNAs of TRC library. **Table S2.** Sequences of 25 barcodes with abnormally increased mapping counts by imperfect matching. **Table S3.** A list of the used options for each tool. **Table S4.** Comparison of mapping results and speed by three tools. (PDF 7691 kb)

## Abbreviations

Barcode-seq: Barcode sequencing
ERG: Early radial glial
GI: Growth inhibition score
MID: Multiplexed ID
MRG: Mid radial glial
NE: Neuroepithelial
NGS: Next-generation sequencing
QC: Quality control
indel: insertions and deletions
TRC: The RNAi Consortium
