# Supplementary material for: Genome-wide functional analysis using the barcode sequence alignment and statistical analysis (Barcas) tool
Source: BMC Bioinformatics. 2016 Dec 23;17(Suppl 17):475. doi: 10.1186/s12859-016-1326-9 (PMC5260075; doi:10.1186/s12859-016-1326-9)
Supplement: Additional file 1: Figure S1. — Pipelines of pooled library screen analysis. Table S1. Public library sets of shRNA, sgRNA and deletion mutant strains. Figure S2. A list of wrong barcodes from 1,230 shRNAs of TRC library. Table S2. Sequences of 25 barcodes with abnormally increased mapping counts by imperfect matching. Table S3. A list of the used options for each tool. Table S4. Comparison of mapping results and speed by three tools. (PDF 7691 kb) [file 12859_2016_1326_MOESM1_ESM.pdf]

Supplementary Online Material

Genome-wide functional analysis using the Barcode Sequence Alignment and Statistical Analysis (Barcas) tool

Jihyeob Mun, Dong-Uk Kim, Kwang-Lae Hoe and Seon-Young Kim

S1. Pooled library screen analysis

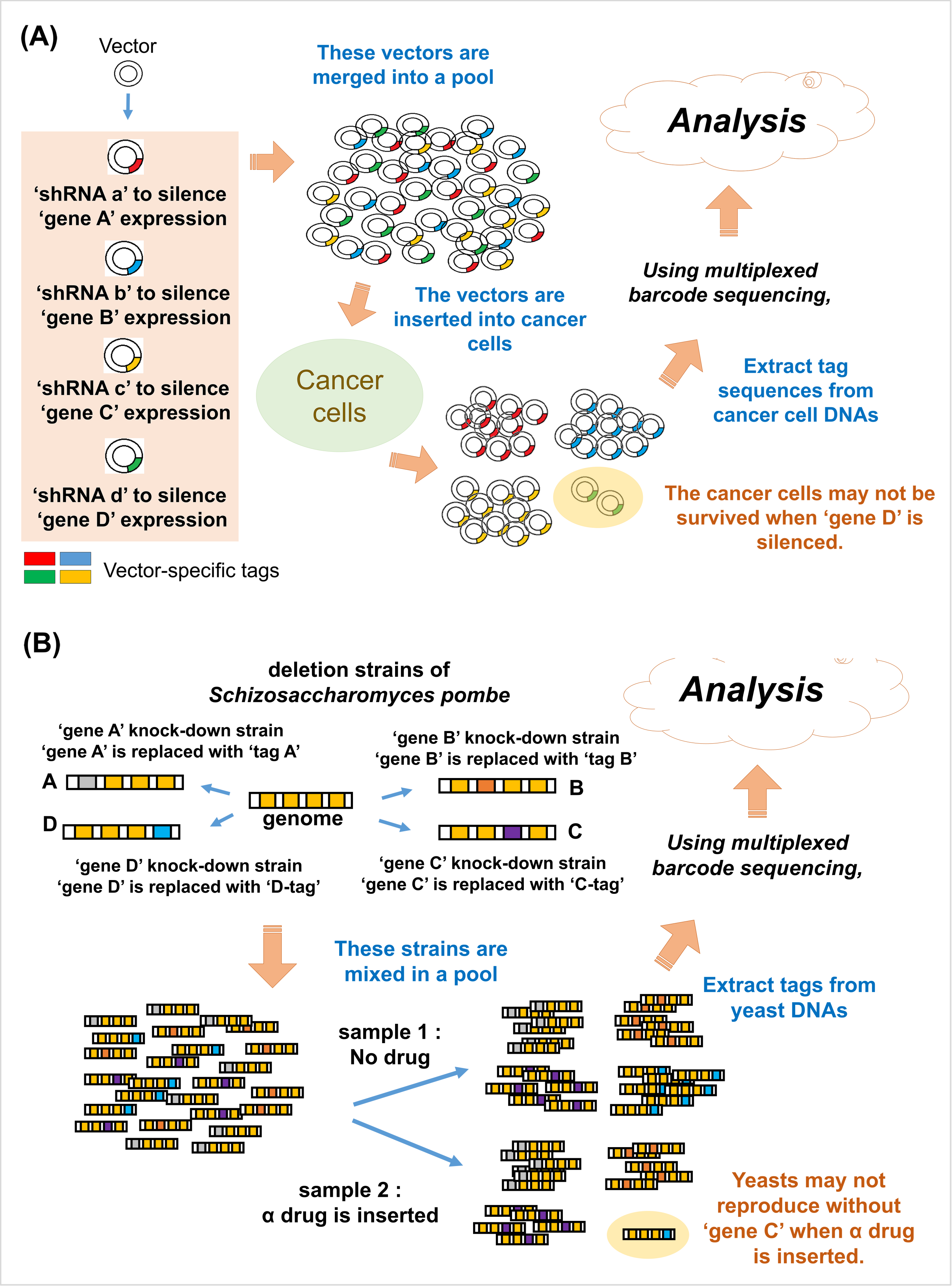

Supplementary Fig. 1. Pipelines of pooled library screen analysis. (A) A pipeline to find target genes for a cancer using shRNAs. Vectors for shRNAs are made to silence a targeted gene expression. The target genes are different by vectors and each vector is distinguished by a shRNA sequence or a tag sequence. All vectors are merged into a pool in the same ratio and the pool is inserted into cancer samples. If some cancer cells having specific shRNA vectors are died, it can be understood that the target gene of the shRNA is related with the cancer survival. To analyze which genes are related with the cancer in a pool, a technique of barcode-seq can be used; (B) A pipeline to find target genes for a drug using barcoded yeast deletion strains. A barcoded yeast deletion strain is a yeast having specific gene knock-down or knock-out. And each strains are distinguished by a tag sequence inserted into the gene sequence loci. Strains having different knock-down or knock-out gene each other are merged into a pool in the same ratio. And, the pool is divided into two, one for a control sample and the other for a case sample. After that, a case sample is treated by a drug. The remained process is the same with (A).

S2. Genome-wide screening libraries tested for sequence similarity

Supplementary Table 1. Public library sets of shRNA, sgRNA and deletion mutant strains. We tested sequence similarity of 11 libraries. In the TRC library, there are 310,119 shRNAs that are too many in a pool. Therefore, we selected a part using vector-ID (selection of pLKO.1), clone-ID, a coding region and human genes. As a result, 61,807 shRNAs were selected.

Screen	Library	Date	Species	Module	Barcode length	Barcode count (unique)	Gene count	Reference
shRNA	TRC	05/Apr/11	*Human*		21-bp	61,621	15,435	http://www.broadinstitute.org/rnai/public/
	Cellecta	15/Feb/12		Module1	18-bp	27,500	5,046	https://www.cellecta.com/
				Module2	18-bp	27,500	5,421	
				Module3	18-bp	27,500	4,923	
sgRNA	yusa		*Mouse*		19-bp	87,437	19,149	Koike et al., 2014
	CeCKOv2	09/Mar/15	*Human*	Library A	20-bp	63,950	21,669	https://www.addgene.org/crispr/libraries/geckov2/
				Library B	20-bp	56,869	19,834	
			Mouse	Library A	20-bp	65,959	22,486	
				Library B	20-bp	61,139	21,263	
Deletion mutant strains	Heterozygous diploid		*Saccharomyces cerevisiae*		20-bp	6,318/UP 6,126/DN	6,131	http://www-sequence.stanford.edu/group/yeast_deletion_project/deletions3.html
			Schizosaccharomyces pombe		20-bp	4,832/UP 4,832/DN	4,832	Kim,D.U. et al, 2010

S3. A list of wrong barcodes among 1,230 barcodes of TRC library

Gene	ID	NE.eCount0	ERG.eCount0	MRG.eCount0	NE.eCount2	ERG.eCount2	MRG.eCount2
PBX2	TRCN0000285144	4,127	3,061	3,597	18,360	13,878	15,604
HOXB2	TRCN0000015562	7,617	3,422	3,581	32,120	24,933	22,662
ATOH8	TRCN0000016993	498	291	204	10,928	8,161	5,854
SKI	TRCN0000010439	9	3	2	2,670	2,039	1,529
TERF2IP	TRCN0000010356	3	1	1	597	395	286
TGIF2	TRCN0000273613	101	48	43	749	491	618
SKI	TRCN0000010437	4	1	0	288	215	177
MYC	TRCN0000010390	1	1	1	165	112	152
JDP2	TRCN0000019000	16	21	11	385	246	217
TFAP2B	TRCN0000019659	41	27	27	513	480	317
NFRKB	TRCN0000014868	53	40	18	436	402	275
EZH2	TRCN0000010475	1	0	1	89	171	89
CSDA	TRCN0000297824	15	7	7	129	72	109
IRX1	TRCN0000021954	157	8	12	173	195	31
NR2F1	TRCN0000350649	32	34	87	228	48	99
KLF13	TRCN0000016925	0	0	0	4	149	0
NCOR2	TRCN0000060706	14	13	13	144	17	14
BCL6	TRCN0000235666	14	14	27	138	17	29
CEBPB	TRCN0000376402	0	1	8	111	36	10
SOX9	TRCN0000342822	28	23	31	171	25	33
MGMT	TRCN0000427322	0	0	0	6	92	2
GBX2	TRCN0000415051	14	21	9	109	22	10
ZIC1	TRCN0000108214	0	1	0	11	94	5
MYBL2	TRCN0000278026	18	14	34	127	19	40
ARX	TRCN0000016335	8	5	11	11	67	12

Mapped read count 0 500 10,000 <=

Supplementary Fig. 2. A list of wrong barcodes from 1,230 shRNAs of TRC library. Control samples, 24 hours after inserting shRNAs without drug treatment in neuropepithelial (NE), early radial glial (ERG) and mid radial glial (RMG) cells, were mapped twice to 1,230 shRNA sequences. First, the data was mapped by perfect matching (eCount0). Second, the data was mapped by imperfect matching allowing two error bases containing mismatches and indels (eCount2). We found 25 (2.03% of 1,230) barcodes whose counts were overwhelmingly increased by imperfect matching. Almost barcodes in the list had few reads with perfect matching and it seems that the original barcodes were mutated. In case of PBX2, HOXB2 and ATOH8, the original as well as mutated barcodes showed significant mapped read counts, suggesting that the original barcodes were designed and synthesized in the right way. However, the imperfect matching results of them contained erroneous barcodes also. The Barcas distinguishes those erroneous barcodes from random PCR or sequencing errors and discards them.

Wrong barcodes can occur by different mechanisms. For example, some erroneous sequences, such as TRCN0000010439 (SKI), are the same in all of the three cells (NE, ERG, MRG) and while other erroneous sequences, such as TRCN0000021954 (IRX1), are found only in a specific cell. We conjecture that errors during barcode synthesis would be responsible for the former cases while errors during insertion or library maintenance would be responsible for the latter cases.

S4. Sequences of wrong barcodes among 1,230 barcodes of TRC

Supplementary Table 2 Sequences of 25 barcodes with abnormally increased mapping counts by imperfect matching. Generally, mismatch or indel errors from PCR or sequencing constitute relatively a small percentage (less than 5%) of mapped reads by imperfect matching and also show a pattern of random distribution of mutant bases across barcode. In contrast, the 25 barcodes below show a pattern of one dominant mutant barcode over the originally designed one, suggesting that those erroneous barcodes didn't come from random PCR or sequencing errors. We designate them as erroneous barcodes distinct from PCR or sequencing errors. Most of the erroneous barcodes showed mismatches, and a few showed insertions and deletions. Currently, it is uncertain how the erroneous barcodes with higher mapped counts (than the original design) were generated and what functions they have. Therefore, the Barcas filters out additional reads from imperfect matching when those erroneous reads show larger mapped count than the mapped count of the original barcode sequence.

Gene	ID	Original.seq	NE.eCount0	ERG.eCount0	NE.eCount0
		Mapped.seq (major one)	NE.eCount2	ERG.eCount2	NE.eCount2
PBX2	TRCN0000285144	ATACTCCCACTTGCAACTATT	4,127 / 18,360 (22.48%)	3,061 / 13,878 (22.06%)	3,597 / 15,604 (23.05%)
		ATACTCCCACTTGTAACTATT	13,177 / 18,360 (71.77%)	9,824 / 13,878 (70.79%)	11,083 / 15,604 (71.03%)
HOXB2	TRCN0000015562	CTTGGATGAAAGAGAAGAAAT	7,617 / 32,120 (23.71%)	3,422 / 24,933 (13.72%)	3,581 / 22,662 (15.8%)
		CTTGGGTGAAAGAGAAGAAAA<br>CTTGGATGAAAGAGAAGAAAA	19,108 / 32,120 (59.49%)	17,626 / 24,933 (70.69%)	15,767 / 22,662 (69.57%)
ATOH8	TRCN0000016993	CTCGTCAATTTCACACGTAAT	446 / 10,928 (4.08%)	218 / 8,161 (2.67%)	204 / 5,854 (3.48%)
		CTCGTCAATTTTACACGTAAT<br>CTCGTCAATTTTACACCTAAT	7,345 / 10,928 (67.21%)	5,417 / 8,161 (66.38%)	3,823 / 5,854 (65.31%)
SKI	TRCN0000010439	GAATCTGCCACTCTCAGAATA	9 / 2,670 (0.34%)	3 / 2,039 (0.15%)	2 / 1,529 (0.13%)
		-AATCTGCCACTCTCAGAATA	2,534 / 2,670 (94.91%)	1,960 / 2,039 (96.13%)	1,441 / 1,529 (94.24%)
TERF2IP	TRCN0000010356	GAGAGTTCTTGCATTGGAACT	2 / 597 (0.34%)	1 / 395 (0.25%)	1 / 286 (0.35%)
		-AGAGTTCTTGCATTGGAACT	551 / 597 (92.29%)	370 / 395 (93.67%)	259 / 286 (90.56%)
TGIF2	TRCN0000273613	CAGGACCCATCACTCCCATTA	101 / 749 (13.48%)	48 / 491 (9.78%)	43 / 618 (6.96%)
		CAGGACCCATCACTCCCCTTA/<br>CAGGGCCCATCACTCCCCTTA	458 / 749 (61.15%)	334 / 491 (68.02%)	452 / 618 (73.14%)
SKI	TRCN0000010437	GATCGAAGACCTGCAGGTGAA	4 / 288 (1.39%)	1 / 215 (0.47%)	0 / 177 (0.0%)
		-ATCGAAGACCTGCAGGTGAA	259 / 288 (89.93%)	199 / 215 (92.56%)	167 / 177 (94.35%)
MYC	TRCN0000010390	GAATGTCAAGAGGCGAACACA	1 / 165 (0.61%)	1 / 112 (0.89%)	1 / 152 (0.66%)
		-AATGTCAAGAGGCGAACACA	156 / 165 (94.55%)	100 / 112 (89.29%)	141 / 152 (92.76%)
JDP2	TRCN0000019000	CGGGAGAAGAACAAAGTCGCA	15 / 385 (3.9%)	21 / 246 (8.54%)	10 / 217 (4.61%)
		CGGGAGAAGAACAAAAACGCA	230 / 385 (59.74%)	141 / 246 (57.32%)	137 / 217 (63.13%)
TFAP2B	TRCN0000019659	CGGTTCTTTCGAGTTTAGTAA	34 / 513 (6.63%)	27 / 480 (5.63%)	26 / 317 (8.2%)
		CGGTTCTTTTGAGTTTTGTAA	163 / 513 (31.77%)	248 / 480 (51.67%)	111 / 317 (35.02%)
NFRKB	TRCN0000014868	CAGGGAGGTTGCATCATTGTT	47 / 436 (10.78%)	38 / 402 (9.45%)	13 / 275 (4.73%)
		CAGGGAGGGTGCATCATTGTT	226 / 436 (51.83%)	143 / 402 (35.57%)	102 / 275 (37.09%)
EZH2	TRCN0000010475	GAAACAGCTGCCTTAGCTTCA	1 / 89 (1.12%)	0 / 171 (0.0%)	1 / 89 (1.12%)
		AAACAGCTGCCTTAGCTTCA/<br>AAACAGCTGCCTTGGCTTCA	86 / 89 (96.63%)	165 / 171 (96.49%)	85 / 89 (95.51%)
CSDA	TRCN0000297824	CGGTTCATCGAAATCCAACTT	15 / 129 (11.63%)	7 / 72 (9.72%)	5 / 109 (4.59%)

		CGGTTCATCGAAAACCAACCT	53 / 129 (41.09%)	37 / 72 (51.39%)	57 / 109 (52.29%)
IRX1	TRCN0000021954	CGACCTGGAAAGCATCGACAT	157 / 173 (90.75%)	8 / 195 (4.1%)	12 / 31 (38.71%)
		CGACCTGGAAAGTATCGACAT	6 / 173 (3.47%)	179 / 195 (91.79%)	2 / 31 (6.45%)
NR2F1	TRCN0000350649	GTCCGCAGGAACTTAACTTAC	32 / 228 (14.04%)	34 / 48 (70.83%)	87 / 99 (87.88%)
		GTCCGCAGGAACTTGACTTAC	174 / 228 (76.32%)	0 / 48 (0.0%)	0 / 99 (0.0%)
KLF13	TRCN0000016925	CGGGCGAGAAGAAGTTCAGCT	0 / 4 (0.0%)	0 / 149 (0.0%)	0 / 0 (NA)
		CGGGCGAGAAGAAGTTCATGGT	3 / 4 (75.0%)	121 / 149 (81.21%)	0 / 0 (NA)
NCOR2	TRCN0000060706	GCAGCGCATCAAGTTCATCAA	14 / 144 (9.72%)	13 / 17 (76.47%)	13 / 14 (92.86%)
		GCAGCGCATCAGGTTCATCAA	122 / 144 (84.72%)	0 / 17 (0.0%)	0 / 14 (0.0%)
BCL6	TRCN0000235666	ACTGCGTTAAAGGCTCGATTT	14 / 138 (10.14%)	14 / 17 (82.35%)	27 / 29 (93.1%)
		ACTGCGTTAAAGGCTCAATTT	116 / 138 (84.06%)	1 / 17 (5.88%)	0 / 29 (0.0%)
CEBPB	TRCN0000376402	ACAAGCACAGCGACGAGTACA	0 / 111 (0.0%)	1 / 36 (2.78%)	8 / 10 (80.0%)
		ACAAGCACAGCGACGAGTACT	99 / 111 (89.19%)	0 / 36 (0.0%)	0 / 10 (0.0%)
SOX9	TRCN0000342822	ACTTCTGAACGAGAGCGAGAA	28 / 171 (16.37%)	23 / 25 (92.0%)	31 / 33 (93.94%)
		ACTTCTGAACGAAAGCGAGAA	122 / 171 (71.35%)	0 / 25 (0.0%)	0 / 33 (0.0%)
MGMT	TRCN0000427322	GAGCAGGGTCTGCACGAAATA	0 / 6 (0.0%)	0 / 92 (0.0%)	0 / 2 (0.0%)
		GAGCAGGGTCTACACGAAATA	6 / 6 (100.0%)	84 / 92 (91.3%)	0 / 2 (0.0%)
GBX2	TRCN0000415051	TGTGGACTACAGCTCGGATGA	14 / 109 (12.84%)	21 / 22 (95.45%)	9 / 10 (90.0%)
		TGTGGACTACAGCTCGGA--A	95 / 109 (87.16%)	0 / 22 (0.0%)	0 / 10 (0.0%)
ZIC1	TRCN0000108214	CGAGCGACAAGCCCTATCTTT	0 / 11 (0.0%)	1 / 94 (1.06%)	0 / 5 (0.0%)
		CGAGCGACAAGCCCTATCTTGT	1 / 11 (9.09%)	79 / 94 (84.04%)	3 / 5 (60.0%)
MYBL2	TRCN0000278026	CTGGCTCTTGACATTGTGGAT	18 / 127 (14.17%)	14 / 19 (73.68%)	34 / 40 (85.0%)
		CTGGCTCTTGACATTGTGAAT	80 / 127 (62.99%)	2 / 19 (10.53%)	0 / 40 (0.0%)
ARX	TRCN0000016335	CTCCTACTGCATCGACAGCAT	8 / 11 (72.73%)	5 / 67 (7.46%)	11 / 12 (91.67%)
		CTCCTACTGCATCGTCAG-AT	1 / 11 (9.09%)	62 / 67 (92.54%)	0 / 12 (0.0%)

S5. Comparison of mapping speed and ratio with two other tools: bowtie and edgeR

We compared the mapping performance of the Barcas with two other tools, bowtie and edgeR. The benchmark conditions are as follows.

- Input: 215 million reads from 4,832 heterozygous diploid deletion strains in *Schizosaccharomyces pombe*.
- Execution environment: 64 GB memory, 6*2.1GHz and Ubuntu 14.0
- MID sequences are 6-bp and 45-bp sequences (barcode + primer) were used as a barcode library.

We compared performance by applying various options due as the three programs support different sets of options. The bowtie supports mismatches (-n option). The edgeR supports mismatches (hairpinMismatchBase option) and position shifts (shiftingBase option). The Barcas supports mismatches, position shifts and indels. Mismatch and indel size is set by eCount option.

Supplementary Table 3. A list of the used options for each tool

Program	Option name	Command
edgeR	Shift0_mis2	allowShifting=FALSE, shiftingBase=0, allowMismatch=TRUE, barcodeMismatchBase=0, hairpinMismatchBase=2, allowShiftedMismatch=FALSE
	Shift1_mis1	allowShifting=TRUE, shiftingBase=1, allowMismatch=TRUE, barcodeMismatchBase=0, hairpinMismatchBase=1, allowShiftedMismatch=TRUE
	Shift2_mis2	allowShifting=TRUE, shiftingBase=2, allowMismatch=TRUE, barcodeMismatchBase=0, hairpinMismatchBase=2, allowShiftedMismatch=TRUE
bowtie	N2	-S -n 2 -l 45 --trim5 6 --norc
Barcas	M0b2	eCount 0 for MID, eCount 2 for barcode, group mapping
	B2	eCount 2 for barcode, group mapping

Supplementary Table 4. Comparison of mapping results and speed by three tools

	edgeR			bowtie	Barcas	
Options	Shift0_mis2	Shift1_mis1	Shift2_mis2	N2	M0b2	B2
Mapped reads with MID (mapping ratio)	161,304,907 (74.96%)	156,229,123 (72.60%)	165,117,873 (76.73%)		182,598,867 (84.86%)	
Mapped reads regardless of MID (mapping ratio)	165,348,327 (76.84%)	164,548,481 (76.46%)	174,509,678 (81.09%)	165,348,327 (76.84%)		192,171,008 (89.31%)
Mapping time	48,755 s	112,195 s	149,953 s	6,420 s	3,458 s	3,655 s

As shown Supplementary Table 4, the Barcas is 1.7 times faster than bowtie and about 13 times faster than edgeR package. Owing to indel mapping, the Barcas mapped at least 8-12% more reads than the other two programs.
